# Neglected tumor in a female with albinism

**DOI:** 10.11604/pamj.2017.28.195.14122

**Published:** 2017-11-01

**Authors:** Fred Bernardes Filho, Andreia de Oliveira Alves

**Affiliations:** 1 Dermatology Division, Department of Medical Clinics, Ribeirão Preto Medical School, University of São Paulo, Ribeirão Preto, Brazil; 2 Medical School, Centro Universitário Barão de Mauá, Ribeirão Preto, São Paulo, Brazil

**Keywords:** Skin neoplasms, basal cell carcinoma, squamous cell carcinoma

## Image in medicine

A 48-year-old female with albinism presented to emergency department with a complaint of a three-day history of nausea and vomiting, accompanied by fever and general weakness. She had a towel wrapped around her left arm and said it was to cover a wound that was growing in size in the last two years. On physical examination, vegetating and exophytic ulcerous tumor on the lateral side of the left arm was observed; there was granulation tissue on all surfaces, release of fluid with pustules and hemorrhagic points. She had a pulse rate of 125/min, blood pressure of 136/78 mmHg, temperature of 38.5 C, (101.3 F) and respiratory rate of 16/min. Laboratory testing was remarkable for elevated white blood cells at 18,230/mm^3^ with left shift and C-reactive protein of 85.4 mg/dl; chest radiography and urine analysis were normal. Biopsy was performed, the diagnosis of giant cutaneous basosquamous cell carcinoma was made and she was referred to oncology department, but her follow-up was lost because she was not found.

**Figure 1:**
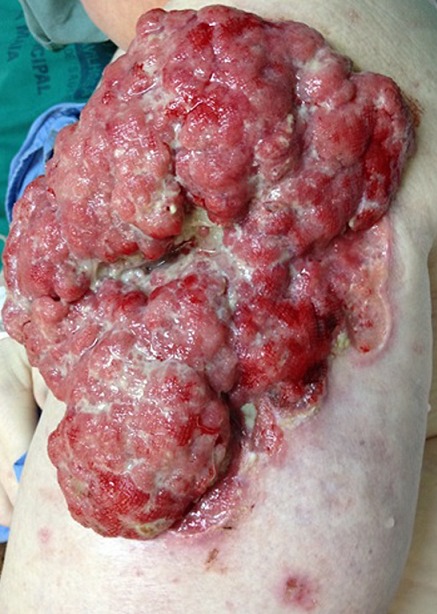
Giant basosquamous cell carcinoma. A bulky, friable tumor on the left arm, measuring 20cm in length x 15cm in width, with elevated and irregular borders

