# Corrigendum: Neglected tumor in a female with albinism

**DOI:** 10.11604/pamj.2018.30.63.16071

**Published:** 2018-05-28

**Authors:** Fred Bernardes Filho

**Affiliations:** 1 Dermatology Division, Department of Medical Clinics, Ribeirão Preto Medical School, University of São Paulo, Ribeirão Preto, Brazil

**Keywords:** Corrigendum, skin neoplasms, basal cell carcinoma, squamous cell carcinoma

## Abstract

This corrigendum corrects article “Neglected tumor in a female with albinism” and its publication references ie The Pan African Medical Journal. 2017;28:195. doi:10.11604/pamj.2017.28.195.14122.

## Corrigendum

The original version of this article [1] had the author’s name wrongly abbreviated on pubmed ie Filho FB instead of Bernardes Filho F.
